# Fatal Hemoperitoneum Due to Rupture of a Hepatic Vascular Malformation With Focal Absence of Hepatic Parenchyma: A Case Report

**DOI:** 10.7759/cureus.96516

**Published:** 2025-11-10

**Authors:** Sayaka Kimoto, Kohichi Tsuneyama, Hirohisa Ogawa

**Affiliations:** 1 Diagnostic Pathology, Tokushima University Hospital, Tokushima, JPN; 2 Pathology and Laboratory Medicine, Tokushima University Graduate School of Biomedical Sciences, Tokushima, JPN

**Keywords:** alcoholic cirrhosis, focal absence of hepatic parenchyma, hemoperitoneum, liver, vascular malformation

## Abstract

We describe an exceptionally rare case of fatal hemoperitoneum resulting from rupture of a hepatic vascular malformation associated with focal absence of hepatic parenchyma. A 60-year-old man with alcoholic cirrhosis died of intra-abdominal bleeding. Autopsy revealed a sharply depressed area on the dorsal surface of the right hepatic lobe. Histologically, this depression corresponded to a complete defect of hepatic parenchyma, replaced only by scant fibro-fatty tissue abutting large portal tracts. Prominent branches of the hepatic artery and portal vein were exposed at the base, accompanied by clusters of dilated CD31-positive vessels and D2-40-positive lymphatics. No tumor was identified. To our knowledge, this is among the first detailed descriptions of a congenital-type hepatic vascular anomaly characterized by focal parenchymal absence, which can culminate in catastrophic hemorrhage.

## Introduction

Vascular malformations of the liver-including hemangiomas, arteriovenous malformations (AVMs), intrahepatic shunts, and congenital anomalies of the portal venous system-are rare and usually silent. Occasionally, they manifest with life-threatening complications such as hemoperitoneum or high-output cardiac failure.

Vascular malformations of the liver are rare, and while pathologists often focus on their structural features, clinicians are concerned about their potential to cause life-threatening complications. For example, spontaneous rupture of hepatic hemangiomas is reported in 1-4% of cases. Ribeiro et al. identified superficial location, large size, and a thin capsule as risk factors for rupture in a series of 28 cases [1]. Pan et al. further highlighted that even hemangiomas <10 cm can rupture when located near the hepatic surface [2].

On the other hand, rupture of other vascular malformations is exceedingly uncommon. Other vascular lesions involve developmental anomalies or shunts. Schmalz et al. reviewed vascular anomalies associated with hepatic shunting, describing arterioportal and AVMs that may distort parenchymal architecture [3]. In congenital absence of the portal vein (Abernethy malformation), areas of parenchymal hypoplasia have been reported adjacent to anomalous vessels [4]. Awareness of such lesions is important not only for pathologists but also for clinicians, as subtle surface changes may carry a fatal risk.

Nevertheless, lesions combining complete absence of hepatic parenchyma with direct exposure of major vessels at a sharply demarcated depression are virtually undocumented. We present a unique autopsy case in which such a malformation caused fatal intra-abdominal bleeding in a cirrhotic liver.

## Case presentation

A 60-year-old man with chronic alcohol abuse had been diagnosed with alcoholic cirrhosis five months before death. There was no clinical history of prior trauma or hepatic surgery. He was admitted with a strangulated umbilical hernia and massive bloody ascites. The CT scan revealed only an umbilical hernia, ascites, and cirrhosis. Soon after admission, he developed cardiopulmonary arrest and died despite intensive resuscitation.

**Figure 1:**
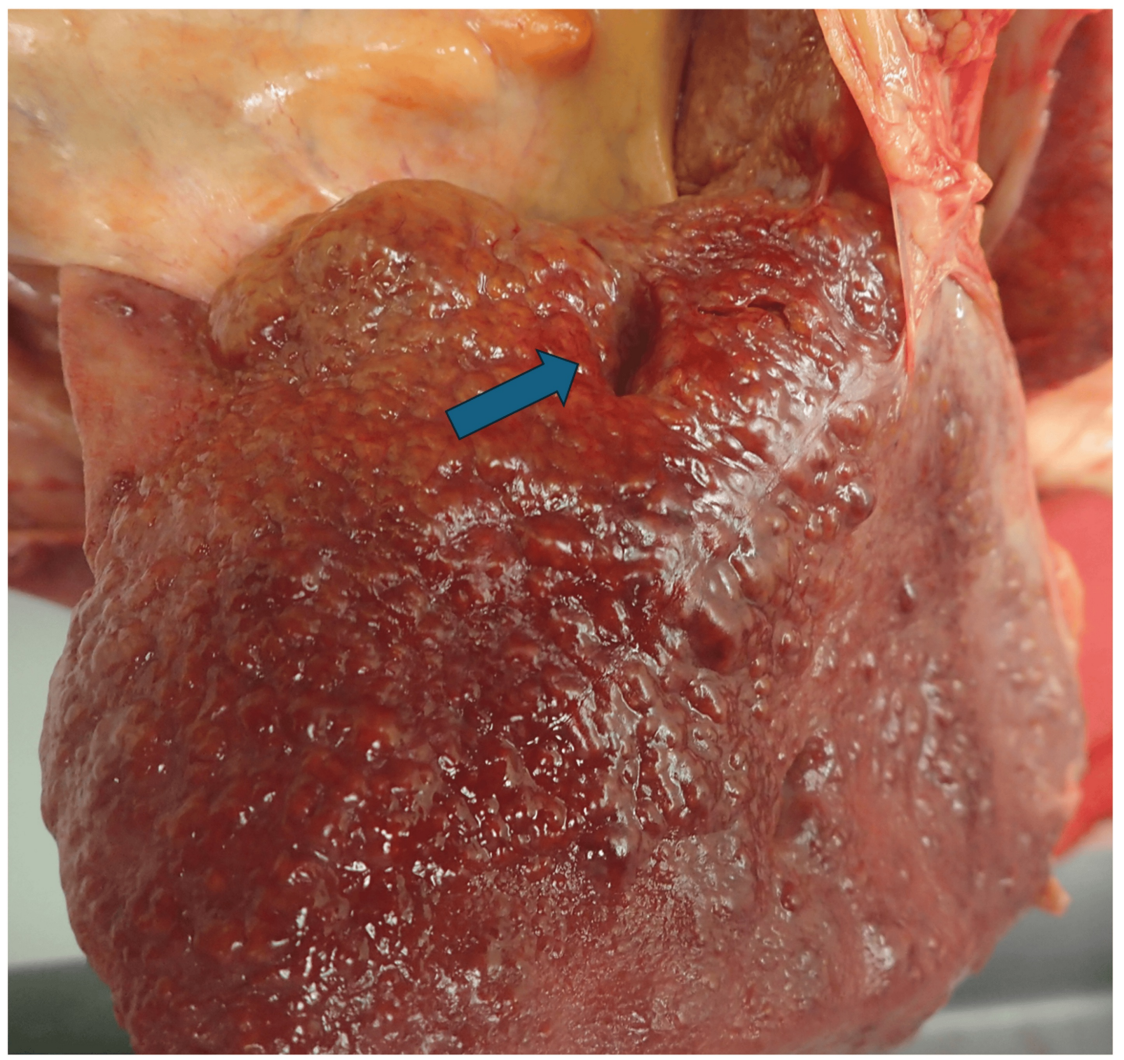
Gross appearance of the liver Gross appearance of the liver showing a sharply demarcated depression on the dorsal surface of the right hepatic lobe.

**Figure 2:**
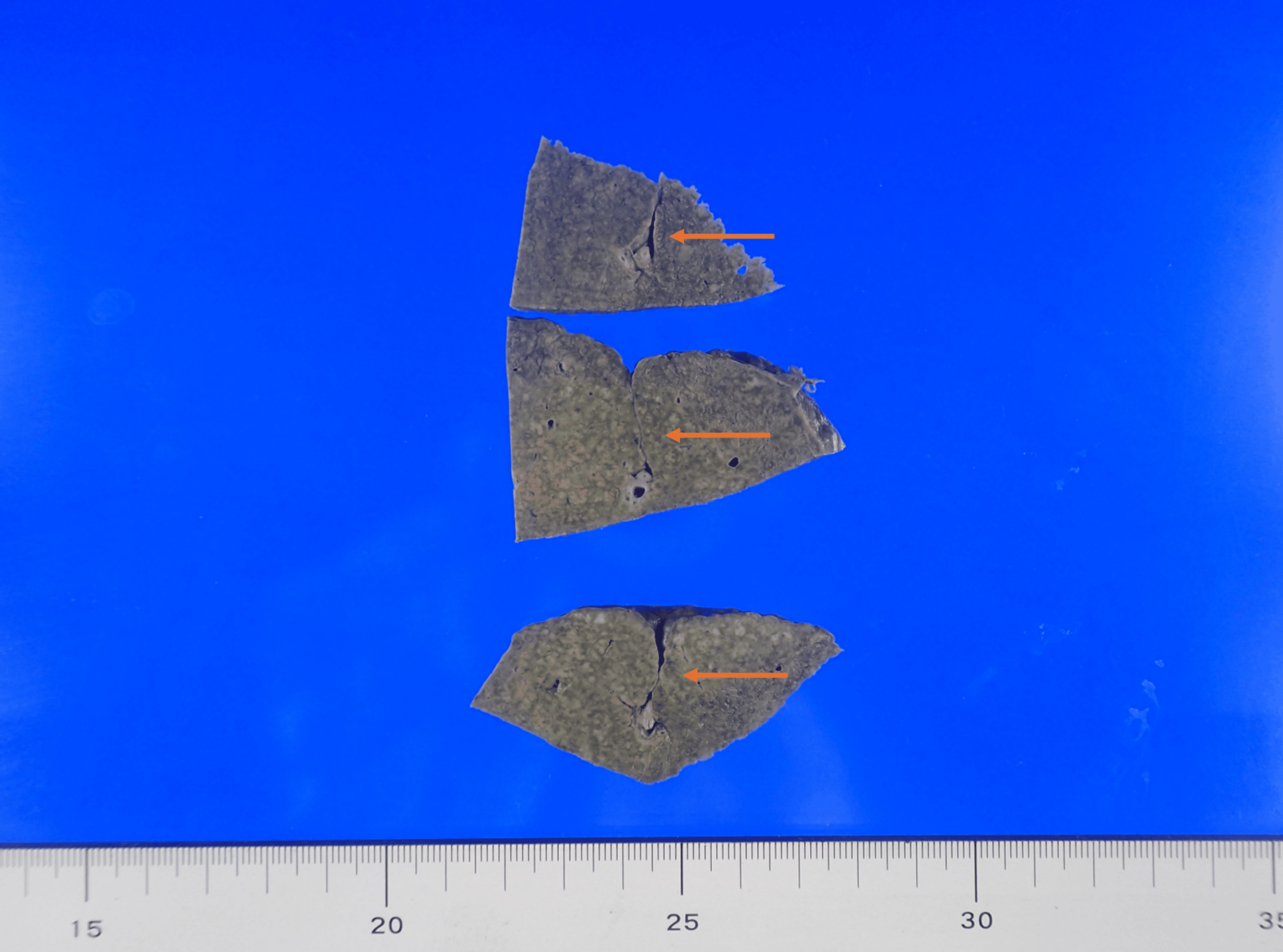
Cut surface Gross image of the hepatic surface showing a sharply demarcated depression (arrow). Unlike usual vascular malformations that remain covered by parenchyma, this lesion corresponds to focal absence of hepatic parenchyma, directly exposing large-caliber vessels at the base. Such structural vulnerability likely predisposed the patient to fatal hemoperitoneum.

At autopsy, the liver weighed 1325 g and exhibited cirrhosis. On the dorsal surface of the right lobe, corresponding approximately to Couinaud segment VI, a sharply demarcated concavity (approximately 20 × 5 mm, extending 20 mm in depth) was noted (Figure 1). On the cut section (Figure 2), the depression extended approximately 20 mm into the parenchyma and contained thick-walled vascular structures.

**Figure 3:**
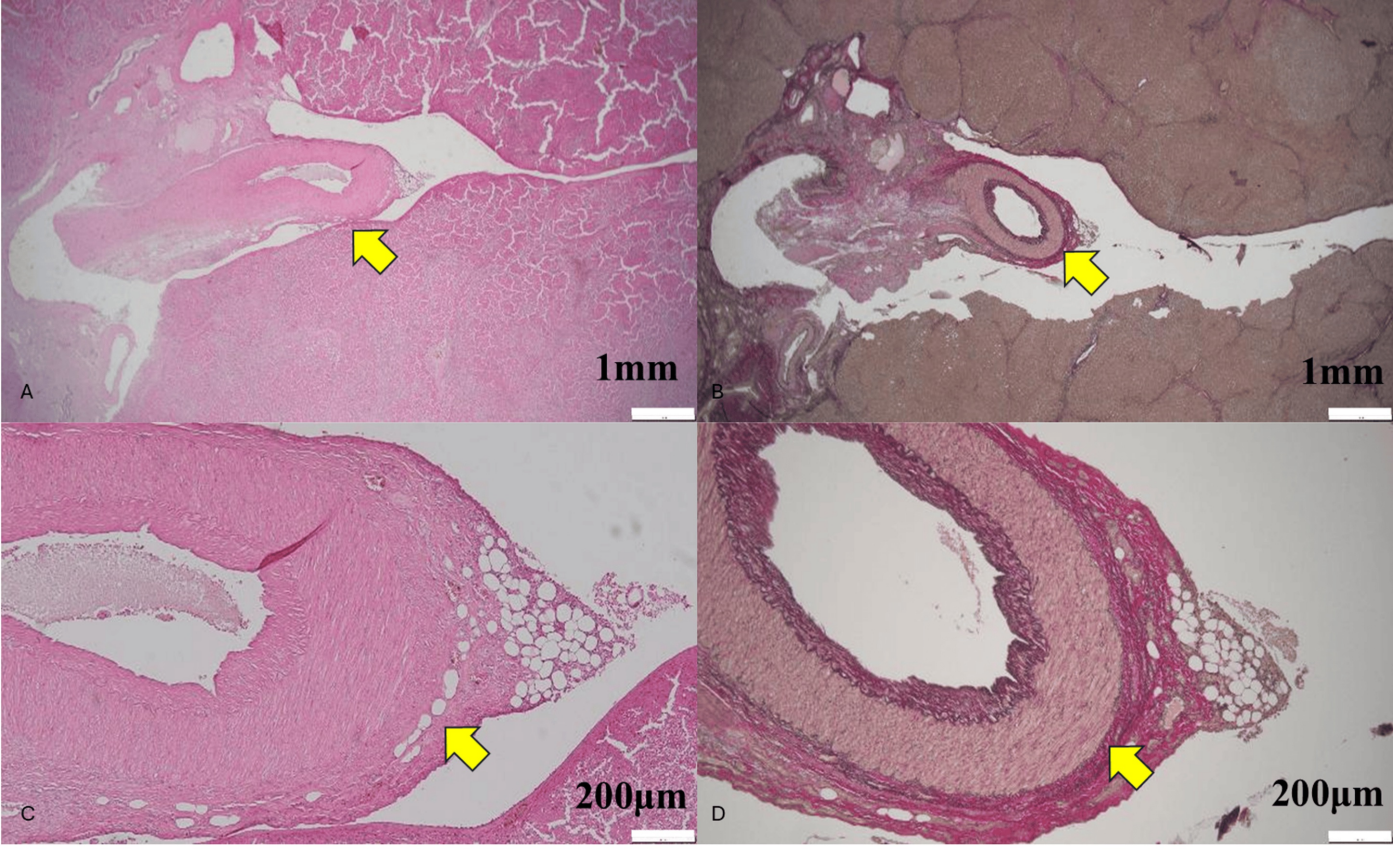
Histological appearance of the lesion (A, C) H&E stain showing abrupt termination of hepatic parenchyma and exposure of a large-caliber artery (arrow) at the base of the depression, with a thin rim of attached fibro-fatty tissue on its adventitial surface. Bar = 1 mm. (B, D) EVG stain of the same area, highlighting the elastic lamina of the exposed artery and confirming the absence of overlying hepatocytes, while also demonstrating a minute amount of adherent fibro-fatty tissue along the vessel wall. Bar = 200 µm.

**Figure 4:**
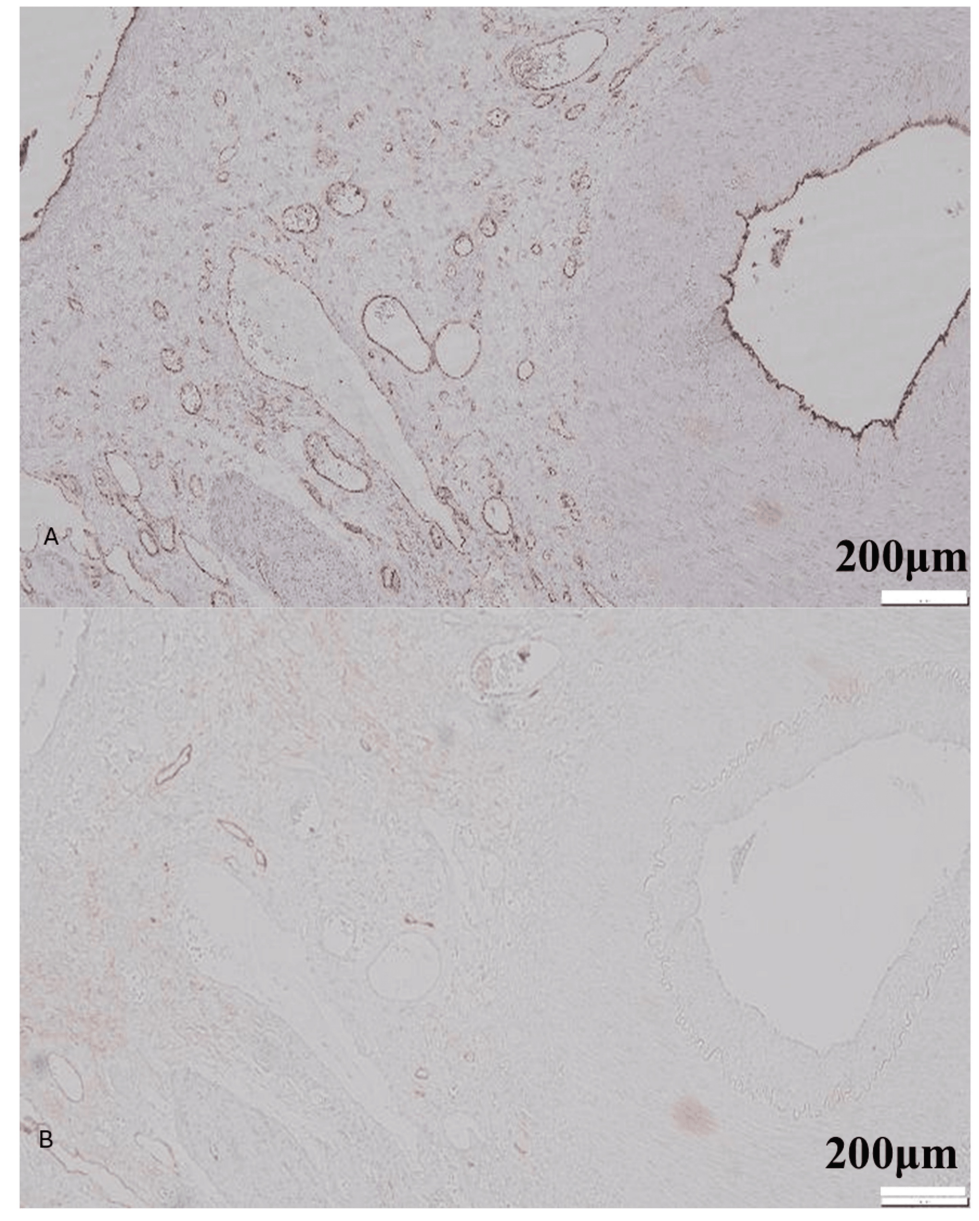
Immunohistochemical characterization of vessels surrounding the exposed artery (A) CD31 staining highlights numerous dilated capillaries (CD31⁺/D2-40⁻) around the vascular complex. Bar = 200 µm. (B) D2-40 staining demonstrates a small population of dilated lymphatic channels (CD31⁻/D2-40⁺) interspersed among the capillaries. Most of the dilated structures represent CD31-positive, D2-40-negative capillaries, whereas a minority correspond to D2-40-positive lymphatic channels. Bar = 200 µm.

Microscopically (Figures 3, 4), the base of the depression lacked hepatocytes, being covered only by scant fibro-fatty tissue. A large hepatic artery and portal vein lie immediately beneath the surface. Elastic Van Gieson staining revealed a thick elastic lamina in the exposed artery. Surrounding stroma showed clusters of dilated CD31-positive vessels and D2-40-positive lymphatics. No neoplastic proliferation was present.

Elsewhere, the liver showed advanced alcoholic cirrhosis with shock-related centrilobular necrosis. Other organs exhibited aspiration pneumonia and strangulated ileus.

## Discussion

This case adds a unique dimension to the spectrum of non-neoplastic vascular anomalies of the liver. Several lines of evidence support the interpretation of a congenital vascular malformation associated with focal parenchymal hypoplasia, rather than an acquired or neoplastic process.

First, the morphology of the lesion was striking: a sharply demarcated concavity with an abrupt termination of hepatic parenchyma. The surface was lined only by scant fibro-fatty tissue, with no hepatocytes or biliary remnants. Such a configuration is inconsistent with post-inflammatory scarring or ischemic atrophy, which typically leaves fibrotic septa, residual cords, or ductular reactions. Similar “parenchymal absence with fibro-fatty tissue” has been alluded to in reports of developmental anomalies of the portal venous system [4].

Second, the exposure of a thick hepatic artery and portal vein at the base of the defect is highly suggestive of a vascular malformation. In embryogenesis, hepatic cords and portal tracts develop concurrently; disruption of this interplay may result in a mature vascular complex devoid of overlying hepatocytes. High-flow malformations, arterioportal or arteriovenous, have been described as retaining large-caliber vessels with minimal parenchymal cover, predisposing to rupture when surface protection is insufficient [3,5].

Third, the presence of dilated CD31-positive channels and D2-40-positive lymphatics surrounding the vascular complex indicates that the anomaly was not restricted to a single vessel, but encompassed a microvascular/lymphatic network. Such remodeling is well documented in hepatic AVMs and congenital shunts, where abnormal flow induces compensatory dilation of sinusoidal or lymphatic channels [3].

Alternative diagnoses were carefully considered. Cavernous hemangioma, the most common benign hepatic vascular lesion, typically remains encapsulated or embedded in parenchyma and does not present as a bare vessel at a surface depression [1]. Abernethy malformation may produce segmental parenchymal hypoplasia but rarely produces a focal defect exposing an artery and portal vein [4]. Traumatic lacerations or infarctions leave necrotic or fibrotic residues rather than a pristine concavity with intact vascular walls.

The mechanism of fatal bleeding in this case is most plausibly explained by the fragility of an exposed arterial branch at the hepatic surface. Even minor physical stress or spontaneous wall failure could trigger massive hemoperitoneum, similar to the pathophysiology proposed for ruptured hemangiomas with scant overlying parenchyma [1,2]. The patient’s background cirrhosis may have increased the fragility of the liver capsule or impaired hemostasis, amplifying the risk of catastrophic bleeding, but cirrhosis alone cannot explain the complete absence of hepatocytes at the lesion site.

From a pathogenetic standpoint, the most plausible hypothesis is a localized defect of hepatocellular colonization around an anomalous vascular complex during organogenesis. This would leave a mature artery and portal vein in situ, with interposed fibro-fatty and lymphatic tissue but no hepatocytes, forming a latent weak point on the liver surface. In schematic terms, typical hepatic vascular malformations remain embedded within preserved parenchyma and are covered by hepatocytes or fibrous tissue, which limits the risk of rupture. By contrast, the present lesion demonstrated a sharply demarcated surface depression with complete absence of hepatocytes, where a large hepatic artery and portal vein were directly exposed, and only scant fibro-fatty tissue was present. This structural vulnerability likely predisposed the patient to fatal hemorrhage.

The lesion likely persisted silently throughout life and became clinically significant only when the vessel wall ruptured. Nevertheless, it remains uncertain whether the lesion arose from a congenital vascular malformation or secondary parenchymal atrophy, and this diagnostic limitation should be acknowledged.

Clinically, fatal hemoperitoneum from a non-tumorous vascular lesion is extremely uncommon, most cases being spontaneous rupture of hemangiomas [1,2]. Recognition of subtle surface anomalies harboring large-caliber vessels is essential for both clinicians and pathologists, especially in patients with cirrhosis, in whom even minor additional insults may precipitate lethal hemorrhage. Awareness of this entity may also refine the interpretation of unexplained intraperitoneal bleeding at autopsy.

## Conclusions

In conclusion, we report a rare hepatic vascular malformation with focal parenchymal absence leading to fatal hemoperitoneum. This case underscores the importance of considering such lesions in unexplained intra-abdominal bleeding and illustrates how structural vulnerability at the liver surface can predispose to rupture. Although the exact etiology remains uncertain, awareness of this possibility may assist both clinicians and pathologists in future cases.
